# Lasting Shifts in Musculoskeletal Injuries Across Pre-, During-, and Post-Pandemic Periods: A Propensity Score-Matched Study

**DOI:** 10.3390/jcm15145441

**Published:** 2026-07-11

**Authors:** Inga Maruszyńska-Małachowska, Ewa Tramś, Kamila Malesa, Rafał Kamiński

**Affiliations:** 1 Department of Physical Education, Józef Piłsudski University of Physical Education in Warsaw, 00-968 Warsaw, Poland; 2 Department of Orthopedics and Musculoskeletal Trauma Surgery, Gruca Orthopaedic and Trauma Teaching Hospital, Centre of Postgraduate Medical Education, Konarskiego 13, 05-400 Otwock, Poland; ewa.trams@gmail.com (E.T.); kama.malesa@gmail.com (K.M.); rkaminski@spskgruca.pl (R.K.)

**Keywords:** traumatology, COVID-19, hospitalization, referral and consultation, demography

## Abstract

**Background**: COVID-19, a global pandemic, has had a profound impact on the world, overwhelming healthcare systems worldwide. These disruptions were associated with significant disparities in healthcare access to routine consultations and elective surgeries, adversely affecting patient health and altering injury patterns in emergency departments. This retrospective observational study aims to compare patient profiles and treatment during the pandemic compared to the pre-pandemic period. **Methods**: Patients admitted to the orthopaedic and traumatology department before, during, and after the COVID-19 pandemic. The full (unmatched) cohort comprised 8413 patients (6175 before, 1188 during, and 1050 after the pandemic). To ensure balanced comparisons, propensity score matching (1:2, based on age, sex, and comorbidities) was applied separately to the during- and after-pandemic groups, each matched against pre-pandemic controls. Collected information incorporated demographics, comorbidities, and injury circumstances. **Results**: After matching, the during-pandemic group (1188 patients) was compared with 2376 pre-pandemic controls, and the after-pandemic group (1050 patients) with 2100 pre-pandemic controls. A higher proportion of same-region re-injuries was observed during and after the pandemic, as well as higher medical leave during the pandemic and post-pandemic, and longer hospital stays of over 10 days during the pandemic and post-pandemic. Injury patterns changed, with an increase in lower leg injuries and a decrease in wrist injuries during the pandemic. Additionally, the proportion of open wounds was lower during the pandemic. **Conclusions**: The pandemic and post-pandemic periods were associated with marked changes in trauma care, including more conservative treatment, fewer surgeries, and longer hospital stays. Same-region re-injuries were more frequent and sports-related injuries less frequent—patterns that temporally coincided with the periods of repeated lockdowns. Notably, differences persisted into the post-pandemic period. Given the retrospective, observational design, these findings represent associations rather than causal effects.

## 1. Introduction

As of October 2024, over 776,618,091 confirmed COVID-19 cases have been reported globally, placing considerable strain on healthcare systems [1]. Countries with high infection rates were compelled to reorganize various medical specialties, including orthopedics, to prioritize emergency response and infection control measures [2,3,4]. This reorganization was associated with reduced access to routine medical consultations and elective surgeries, adversely affecting the overall health of patients, particularly those with chronic conditions [4,5,6]. The global adaptation of telemedicine in orthopedic and trauma departments has received positive feedback from patients [7,8].

While the number of patients who visited emergency departments during the COVID-19 pandemic significantly decreased, the profile of those visiting orthopedic emergency rooms shifted, reflecting changes in the types of injuries sustained during lockdowns [9]. A decrease in the overall number of fractures was noted compared to the pre-pandemic period, including reductions in open lower limb fractures and wrist fractures. A significant reduction was also observed in referrals for simple fractures, native joint dislocations, wounds, and soft tissue injuries. Among pediatric patients, a similar decline in referrals for simple fractures was noted [10,11,12,13].

In addition to altering patient profiles, the pandemic period was associated with changes in the availability of care and treatment approaches. The overall number of surgeries declined, associated with an increase in non-surgical treatments of fractures. An extension in the time from injury to surgical treatment was also observed among patients with open fractures [14]. The length of hospital stays nearly doubled, and complications associated with mortality rates increased [13,15,16,17]. The literature indicates a higher mortality rate associated with fractures during the pandemic [12,18].

However, road traffic accidents remained the leading injury mechanism throughout the pre-pandemic and pandemic periods; there was an increase in cases of falls from heights and work-related injuries during the pandemic, while injuries occurring in sports settings were notably lower than before the pandemic. Additionally, an increase in bicycle-related accidents was also observed [10,11,12].

This study examines changes observed at our hospital that coincided with global changes in healthcare systems. The purpose of this retrospective observational study was to compare patient profiles and treatment methods during the pandemic with those of similar groups of patients treated at our facility both prior to the pandemic and after its resolution. The study considered factors such as patients’ sex, age, comorbidities, injury recurrence (primary or re-injury of the same region), injury location according to ICD-10 codes, and the context of the injury (work-related, daily life, or sports-related, with a focus on various sports disciplines). Integrating demographic shifts, variations in injury patterns, healthcare access, and changes in treatment will help to understand and modify future healthcare services.

It should be noted that this study evaluates hospitalized patients from a single orthopaedic trauma centre; the observed changes may therefore reflect shifts in admission practices, referral thresholds, resource allocation, or institutional policy, in addition to—or independently of—any true epidemiologic changes in injury occurrence in the wider population.

## 2. Materials and Methods

### 2.1. Study Design

**Figure 1:**
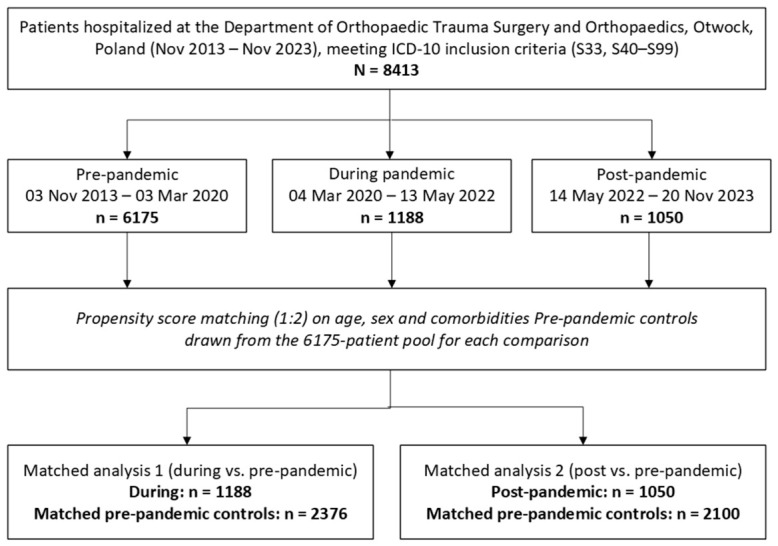
STROBE flow diagram of patient selection and cohort formation. The full unmatched cohort comprised 8413 hospitalizations meeting the ICD-10 inclusion criteria across the three study periods. Propensity score matching (1:2, on age, sex, and comorbidities) was performed separately for the during- and post-pandemic groups, each matched against controls drawn from the pre-pandemic pool (*n* = 6175), yielding 1188 vs. 2376 (matched analysis 1) and 1050 vs. 2100 (matched analysis 2).

This study was designed as a retrospective cohort analysis, conducted in accordance with the Strengthening the Reporting of Observational Studies in Epidemiology (STROBE) guidelines [19]. All hospitalizations at the orthopedic trauma center during the study period were included in three periods: pre-pandemic (3 November 2013–3 March 2020), during-pandemic (4 March 2020–13 May 2022), and post-pandemic (14 May 2022–20 November 2023) the COVID-19 pandemic (Table 1). The study period was divided into three intervals to capture operationally distinct phases of the pandemic’s impact on care delivery. The pre-pandemic period (3 November 2013–3 March 2020) served as the baseline. The pandemic period began on 4 March 2020, the date of the first laboratory-confirmed SARS-CoV-2 case in Poland, after which orthopaedic and trauma services were reorganized and elective care restricted. The post-pandemic period began on 14 May 2022, following the Council of Ministers’ regulation of 13 May 2022 that ended the nationwide state of epidemic. This structure allowed us to compare baseline injury patterns and treatment with those during active pandemic restrictions and to assess whether they subsequently returned to pre-pandemic levels. Propensity score matching was utilized to control for confounding variables and ensure balanced comparison between the two groups. The process of patient selection and cohort formation is summarized in Figure 1.

### 2.2. Data Sources

The data source for this study was the medical database of hospitalized patients, managed using the AMMS (Asseco Medical Management Solutions, Warsaw, Poland) system. The data were exported from the AMMS system via a SQL query and subsequently saved in an XLS spreadsheet for further analysis. All ICD-10 classification included injuries in the musculoskeletal system treated by orthopedic surgery system were included: S33, S40–S49, S50–S59, S60–S69, S70–S79, S80–S89, S90–S99.

### 2.3. Setting and Participants

The study was conducted at the Department of Orthopedic Trauma Surgery and Orthopedics, Centre of Postgraduate Medical Education, located at the Gruca Orthopedic and Trauma Teaching Hospital in Otwock, Poland. The data analyzed covered all hospitalizations within this orthopedic trauma center from 2013 to 2023, providing a comprehensive overview of injury cases over ten years.

### 2.4. Variables

The collected information included: demographics—sex, age, economic category (pre-production, production, post-production), and biological category (children, adults, elderly); health characteristics—co-morbidities and injury recurrence (primary vs. re-injury of the same region); treatment-related data—surgical intervention, medical leave, and length of hospitalization (<4, 5–10, >10 days); injury coding—ICD-10 codes for anatomical localization and injury type (International Statistical Classification of Diseases and Related Health Problems) [20]; circumstances of injury—work-related, daily life, or sports-related; and, for sports injuries, the specific activity (gymnastics, football, climbing, water sports, cycling, skiing, and others).

A re-injury of the same region was defined as a repeat presentation by the same patient for an injury localized to the same anatomical region (ICD-10 S-code localization), irrespective of the specific diagnosis; the first presentation for a given region was classified as primary. This category, therefore, reflects the affected anatomical region rather than an exact diagnostic match, and applies only to patients presenting on a subsequent occasion.

### 2.5. Statistical Analysis

A total of 8413 patients were included in this study, with 1188 patients hospitalized during the pandemic. The pre-pandemic cohort initially included 6175 patients. Propensity score matching (PSM) was performed based on age, sex, and comorbidities, yielding a matched sample of 2376 patients hospitalized before the pandemic. Similarly, for the 1050 patients treated post-pandemic, PSM was conducted to obtain a matched sample of 2100 patients from the pre-pandemic cohort.

To reduce the influence of potential confounding, propensity score matching (PSM) was performed separately for the pre-pandemic versus during-pandemic comparison and for the pre-pandemic versus post-pandemic comparison. Propensity scores were estimated using logistic regression based on age, sex, hypertension, osteoporosis, and diabetes mellitus. In each comparison, patients from the during-pandemic or post-pandemic period were matched with patients from the larger pre-pandemic group using greedy nearest-neighbour matching in a 1:2 ratio, without replacement. No caliper was used in the primary analysis. Covariate balance was assessed before and after matching using standardized mean differences (SMDs). An absolute SMD below 0.10 was considered to indicate adequate balance. Full technical details of the PSM procedure, balance tables, and Love plots are provided in the Supplementary Materials. The analyses used tests of percentage comparisons between the two groups.

Because the analysed periods differed in duration, admission rates were calculated per month and per year. Admission rates during the pandemic and post-pandemic periods were compared with the pre-pandemic period using incidence rate ratios (IRRs) with 95% confidence intervals. Temporal changes were presented using monthly admission counts and a three-month moving average, including complete calendar months only.

Quantitative statistical analysis was performed using R™ software 4.4.1 (R Foundation for Statistical Computing) [20]. The R Studio user interface version 2023.03.1 was used. The analysis utilized the “tidyverse” package (version 2.0.0) for data manipulation and visualization, and the “gtsummary” package (version 1.7.2) for generating summary statistics. Statistically significant level was established as *p*-value < 0.05. Descriptive statistics were expressed as follows. Continuous variables were expressed as mean (standard deviation), median, and 25th and 75th percentiles ([25–75%]). Differences between groups were compared using the Mann–Whitney test in the case of continuous variables. Categorical (qualitative) variables were expressed as absolute (*n*) and relative (%) frequencies. Comparison of categorical variables between groups was performed using the Fisher test. *p*-values were adjusted for multiple comparisons using the Benjamini–Hochberg false discovery rate, and q-values are reported alongside unadjusted *p*-values. For dichotomous variables, effect sizes were additionally reported as risk differences in percentage points with 95% confidence intervals.

### 2.6. Equity, Diversity, and Inclusion Statement

Due to the retrospective nature of the study, all patients admitted to the orthopedic and traumatology department during the specified periods were included, and no recruitment strategy was applied. Therefore, planning for diversity in participant inclusion was not applicable. However, the analysis accounted for differences in age and sex.

The investigator and author team is gender-balanced and includes individuals at various stages of their academic careers. The team represents diverse professional experiences and perspectives.

Data analysis and interpretation considered relevant inequities, including sex-related differences. Limitations related to generalizability due to the lack of detailed information on certain marginalized groups have been acknowledged in the discussion.

## 3. Results

The analysis began by standardising the number of admissions to the duration of each observation period. During the pre-pandemic period, 6175 admissions were recorded over 76.0 months, corresponding to 81.3 admissions per month and 975.1 admissions per year. During the pandemic period, 1188 admissions were recorded over 26.3 months, corresponding to 45.1 admissions per month and 541.7 admissions per year. The admission rate was significantly lower than in the pre-pandemic period (IRR = 0.56; 95% CI: 0.52–0.59; *p* < 0.001).

During the post-pandemic period, 1050 admissions were recorded over 18.3 months, corresponding to 57.5 admissions per month and 689.8 admissions per year. Although the admission rate increased compared with the pandemic period, it remained significantly lower than in the pre-pandemic period (IRR = 0.71; 95% CI: 0.66–0.76; *p* < 0.001) (Supplementary Table S4).

The analysis of monthly admission counts demonstrated substantial temporal variability. Admissions declined after the onset of the pandemic and partially recovered in the subsequent months. During the post-pandemic period, monthly admission counts were higher than during the lowest months of the pandemic period but did not return to the average level observed before the pandemic (Supplementary Figure S3).

**Figure 2:**
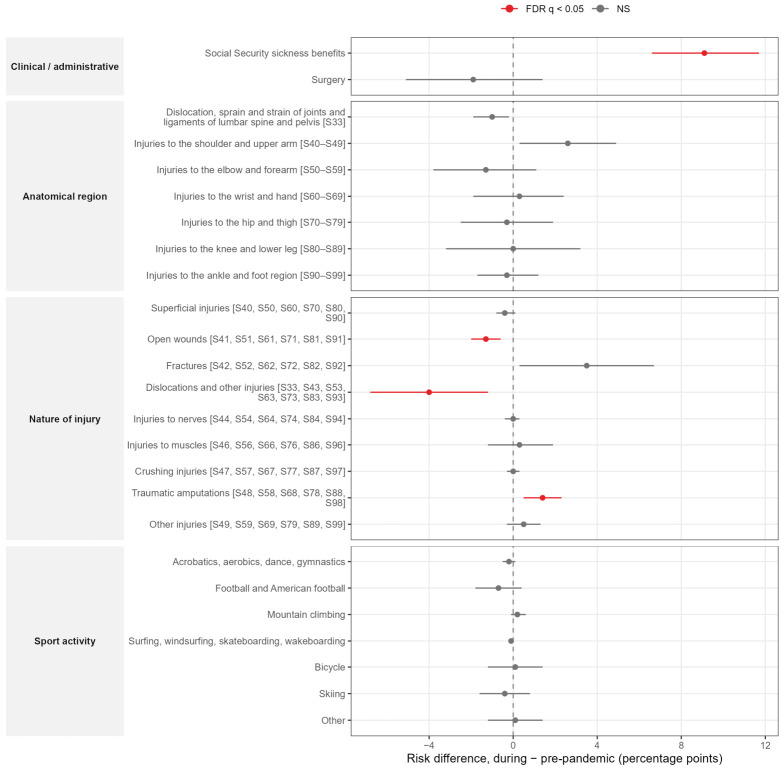
Risk differences in musculoskeletal injury characteristics, treatment and circumstances during versus before the COVID-19 pandemic. Note: Points represent the risk difference (during-pandemic minus pre-pandemic, in percentage points) with 95% confidence intervals derived from cluster-robust variance accounting for the matched design; shown for dichotomous variables only. The dashed line at zero indicates no difference between periods. Estimates significant after false discovery rate correction (Benjamini–Hochberg, *q* < 0.05) are highlighted in red.

**Figure 3:**
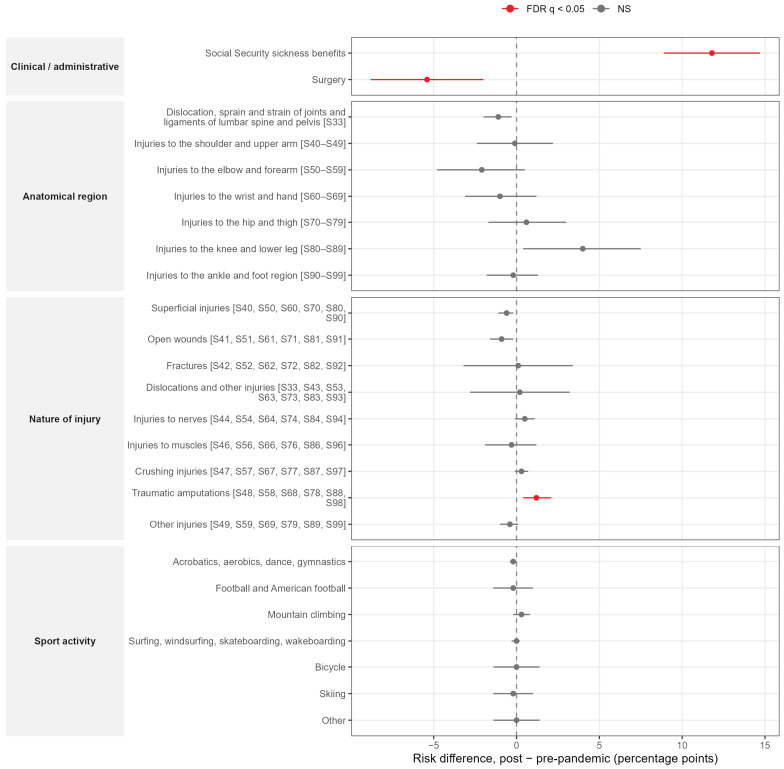
Risk differences in musculoskeletal injury characteristics, treatment and circumstances after versus before the COVID-19 pandemic. Note: Points represent the risk difference (post-pandemic minus pre-pandemic, in percentage points) with 95% confidence intervals derived from cluster-robust variance accounting for the matched design; shown for dichotomous variables only. The dashed line at zero indicates no difference between periods. Estimates significant after false discovery rate correction (Benjamini–Hochberg, *q* < 0.05) are highlighted in red.

Matched data on pre-pandemic (*n* = 2376) and during-pandemic (*n* = 1188) hospitalizations are presented in Figure 2; matched data on pre-pandemic (*n* = 2100) and post-pandemic (*n* = 1050) hospitalizations are presented in Figure 3. The two pre-pandemic groups differ in size because each comparison involved a separate propensity score matching procedure (1:2) drawing controls from the same unmatched pre-pandemic pool of 6175 patients.

During the pandemic, re-injury of the same anatomical region predominated over primary injury (94.1% vs. 80.7% before the pandemic; *p* < 0.001), patients more frequently used Social Security sickness benefits (21.3% vs. 12.2%; risk difference [RD] +9.1 percentage points [pp], 95% CI 6.6 to 11.7; *q* < 0.001), and hospital stays were longer, with a higher proportion exceeding 10 days (24.3% vs. 19.9%; overall *p* = 0.006). The proportion of patients undergoing surgery did not differ significantly between periods (68.9% vs. 70.8%; *p* = 0.26). Regarding the nature of the injury, open wounds were less frequent during the pandemic (0.5% vs. 1.8%; RD −1.3 pp, 95% CI −2.0 to −0.6; *q* = 0.008), as were dislocations and other injuries (20.9% vs. 24.9%; RD −4.0 pp, −6.8 to −1.2; *q* = 0.039), whereas traumatic amputations were more frequent (2.1% vs. 0.7%; RD +1.4 pp, 0.5 to 2.3; *q* = 0.003). After correction for multiple comparisons (Benjamini–Hochberg), none of the ICD-10 anatomical-region categories differed significantly between periods, and the distribution of injury circumstances (daily life/work vs. sport) was unchanged (*p* = 0.26).

The post-pandemic sample showed a broadly similar pattern relative to the pre-pandemic period. Re-injury of the same region predominated to an even greater extent (95.2% vs. 82.9%; *p* < 0.001), the use of sickness benefits approximately doubled (24.5% vs. 12.7%; RD +11.8 pp, 95% CI 8.9 to 14.7; *q* < 0.001) and—in contrast to the during-pandemic period—surgery was performed significantly less often (65.1% vs. 70.5%; RD −5.4 pp, −8.8 to −2.0; *q* = 0.013). Hospital stays exceeding 10 days remained more frequent post-pandemic (27.5% vs. 20.2%; overall *p* < 0.001). Among injury types, only traumatic amputations differed significantly after FDR correction, being more common post-pandemic (1.8% vs. 0.6%; RD +1.2 pp, 0.4 to 2.1; *q* = 0.012). The nominal reductions in open wounds and superficial injuries and the nominal increase in lower-leg (S80–S89) injuries did not survive correction for multiple comparisons, and the distribution of injury circumstances did not change (*p* = 0.44).

Table 2 presents hospitalization length stratified by treatment type for the before–during comparison (pre-pandemic *n* = 2376). The length of hospitalization and its relationship with surgical and conservative treatments also revealed significant differences. During and after the pandemic, among surgically treated patients, 75–76% had short stays (0–4 days), compared to only 58% before the pandemic. In the pre-pandemic period, a higher percentage of patients (31%) were hospitalized for 5–10 days compared to 20% during and 19% after the pandemic. Conversely, hospital stays exceeding 10 days were more common in patients treated conservatively during and after the pandemic (67–70%) than before (27%), in contrast to the pre-pandemic pattern, where longer stays were less frequent among conservatively treated patients.

These findings show that injury patterns, treatment modalities, and hospitalization dynamics differed during and after the pandemic compared with the pre-pandemic period, with differences persisting after its resolution.

### Gender Variables

After matching cases of injuries during the pandemic with cases of injuries that occurred before the pandemic, the results for the group of women are as follows. During the pandemic, female patients significantly more often experienced re-injuries of the same anatomical region. They underwent surgery less frequently. Their hospitalization generally lasted longer. However, no significant differences were detected in the circumstances of the injuries. (Supplementary Tables S4 and S6).

In terms of the types of injuries, during the pandemic, women were statistically significantly more often admitted with injuries categorized as S40–S49 (*p* = 0.02), S80–S89 (*p* = 0.04), and dislocation and other injuries (*p* = 0.046). Interestingly there were fewer admissions for S50–S59 (*p* = 0.009) and open wounds (*p* = 0.01).

Similarly, after matching cases of injuries during the pandemic with cases of injuries that occurred before the pandemic, the results for the group of men are as follows. During the pandemic, male patients also significantly more often experienced re-injuries of the same anatomical region. They underwent surgery less frequently. Their hospitalization generally lasted longer. However, in contrast to women, significant differences were detected in the circumstances of the injuries—during the pandemic, men were less frequently hospitalized due to sports-related injuries. In terms of the types of injuries, during the pandemic, men were statistically significantly more often admitted with injuries categorized as S80–S89 (*p* = 0.02), traumatic amputations (*p* = 0.03), and other injuries (*p* < 0.001); there were significantly fewer admissions with S60–S69 (*p* = 0.006) and open wounds (*p* < 0.001) (Supplementary Tables S5 and S7).

## 4. Discussion

The healthcare system faced significant challenges during and after the COVID-19 pandemic, requiring rapid adaptation to unprecedented circumstances. Access to medical services was limited or, in many cases, canceled, particularly in public healthcare facilities [21]. Although telemedicine expanded in primary care, its applicability to trauma patients remained limited due to the specificity and immediacy of their treatment needs [7]. Additionally, the need to reduce hospitalizations and repurpose many departments for COVID-19 care placed further strain on the availability of resources for trauma patients [6,22]. This study aimed to evaluate changes in hospitalization duration, medical leave, and injury types associated with the pandemic. By comparing data from before, during, and after the pandemic, the study sought to provide insights for improving future medical services.

Our findings indicated a higher prevalence of re-injuries compared to new fractures during the pandemic, alongside a reduced number of surgical procedures. This pattern is consistent with the postponement of non-urgent cases in favor of conservative treatment due to multiple factors. These included patient fears of COVID-19 transmission in healthcare settings, the repurposing of hospitals into critical care units, and the redeployment of surgeons to treat COVID-19 patients [23,24]. Longer hospital stays observed during the pandemic may reflect increased complexity of trauma cases, reduced availability of operating rooms, and fewer surgical staff. In addition, other biases, such as interrupted rehabilitation and delayed follow-up, should be considered according to COVID pandemic.

Sports-related injuries significantly decreased during the lockdown period, consistent with the closure of schools, sports clubs, and restrictions on physical activities implemented by governments [25]. Similar findings were reported by Sephton et al. [11], who analyzed orthopedic referrals and surgical procedures during the 2020 lockdown compared to 2019. They observed a 35.3% reduction in emergency department visits, a significant drop in sports-related injuries, and a 38.8% decline in surgical procedures. Interestingly, they also noted a reduction in wrist surgeries during the lockdown. Since wrist fractures predominantly affect older women—a high-risk group with low functional demands—hospital admissions for these cases were carefully reconsidered to minimize infection risks. Studies investigating conservative treatment for wrist fractures during the pandemic reported favorable outcomes, even for intra-articular fractures [26,27].

Interestingly, post-pandemic trends in hospital usage and treatment approaches remained consistent with those observed during the pandemic. A possible explanation is that patients continued to avoid emergency departments unless necessary, possibly reflecting lingering concerns about COVID-19 exposure. Additionally, physicians may have adopted a preference for conservative treatment approaches, reducing the need for immediate hospital admission. In our study, upper limb injuries were lower during the pandemic and post-pandemic period, unlike lower leg injuries, which were higher. As the study includes only patients admitted to hospital, those findings could reflect changes in injury occurrence caused by the pandemic, as well as changes only in admittance to the hospital. Patients may avoid the emergency department if it is not necessary due to inability to walk, or non-surgery treatment being chosen over admittance to the hospital; however, in our study we did not evaluate patients’ adjustment, triage decision or clinician decisions in the ED.

### 4.1. Clinical Implications

These findings underscore the lasting impact of the COVID-19 pandemic on healthcare delivery, highlighting changes in patient behavior, treatment strategies, and resource allocation that persisted even after the acute phase of the crisis. By understanding these shifts, healthcare systems can better prepare for future public health emergencies and optimize care delivery in challenging circumstances.

### 4.2. Strengths and Limitations

There are several strengths in this study. First of all, there is a large population sample, including data from the largest orthopedic trauma hospital in Poland, providing a comprehensive sample size that improves the reliability of results. Besides this, there is detailed data collection, including demographics, gender, injury types, treatment details, and hospitalization trends, providing a multifaceted analysis. In addition, by analyzing three distinct time periods (pre-pandemic, during the pandemic, and post-pandemic), the study effectively highlights trends and changes in patient profiles and treatment strategies. Using propensity score matching minimized confounding variables, ensuring fair comparisons across the periods.

This study has several limitations that should be considered when interpreting the findings. The retrospective, single-centre design restricts both the generalisability of results—as findings from a tertiary orthopaedic trauma hospital may not reflect smaller or non-specialist centres across Poland—and the ability to establish causal relationships; all observed differences should therefore be interpreted as associations. Data were derived from existing administrative records, and ICD-10 codes were assigned as part of routine clinical documentation, meaning that coding inaccuracies or changes in coding practice over the ten-year study period cannot be excluded. The re-injury classification likewise relied solely on records held at our institution, so patients initially treated elsewhere may have been misclassified as primary presentations.

Beyond pandemic-related factors, organisational changes at the study centre—such as shifts in staffing, departmental capacity, or referral pathways—may have independently contributed to the observed patterns and cannot be fully disentangled from the effects of the pandemic itself. Although propensity score matching on age, sex, and comorbidities reduced the influence of known confounders, residual confounding from unmatched variables such as injury severity or socioeconomic status cannot be excluded; additionally, as the pre-pandemic pool served as the control group for both the during- and post-pandemic comparisons, some dependency between the two analyses may exist. Finally, the unequal duration of the three study periods may affect the comparability of trends across time. Additionally, only hospitalized patients were analyzed. During and after the pandemic, minor injuries may have been treated conservatively, and this could have an impact on results.

## 5. Conclusions

This retrospective observational study demonstrated that specific musculoskeletal injury patterns, treatment approaches, and hospitalization dynamics were associated with the COVID-19 pandemic period, with differences persisting beyond its resolution. Surgical rates were lower, hospital stays were longer, and same-region re-injuries were more frequent during and after the pandemic, while sports-related injuries declined and lower leg injuries became more prevalent. As findings derive from a single-centre retrospective study, they should be interpreted as associations rather than causal effects, and multi-centre research is needed to confirm whether these trends reflect broader changes in trauma care delivery.

## Figures and Tables

**Table 1:** Demographic characterization of the cohorts.

	Pre-Pandemic *n* = 6175	During-Pandemic *n* = 1188	Post-Pandemic *n* = 1050
Woman	2579 (42%)	493 (41%)	491 (47%)
Man	3596 (58%)	695 (59%)	559 (53%)
Mean age [mean (SD)]	43.93 (22.46)	40.21 (20.34)	42.77 (20.20)
**Economic age**			
Pre-production	896 (15%)	226 (19%)	148 (14%)
Production (18–64 male, 18–59 female)	3881 (63%)	777 (65%)	700 (67%)
Post-production	1398 (23%)	185 (16%)	202 (19%)
**Biological age**			
Children (0–18 yr.)	673 (11%)	154 (13%)	89 (8%)
Adults (18–65 yr.)	4304 (70%)	896 (75%)	797 (76%)
Elderly (>65 yr.)	1198 (19%)	138 (12%)	164 (16%)
**Comorbidities**			
Hypertension	1058 (17%)	168 (14%)	182 (17%)
Osteoporosis	66 (1.1%)	10 (0.8%)	20 (1.9%)
Diabetes	371 (6.0%)	109 (9%)	63 (6.0%)

**Table 2:** Length of hospitalization (days) pre-, during-, and post-COVID-19 pandemic, stratified by treatment type (surgery vs. conservative management).

	PRE-PANDEMIC *n* = 2376	DURING-PANDEMIC *n* = 1188	POST-PANDEMIC *n* = 1050	*p*-Value
**All**				
0–4	1398/2376 (59%)	712/1188 (60%)	618/1050 (59%)	<0.001
5–10	653/2376 (27%)	187/1188 (16%)	143/1050 (14%)	
>10	325/2376 (14%)	289/1188 (24%)	289/1050 (28%)	
**Surgery**				
0–4	1124/1911 (58%)	612/819 (75%)	523/684 (76%)	<0.001
5–10	585/1911 (31%)	166/819 (20%)	127/684 (19%)	
>10	202/1911 (11%)	41/819 (5%)	34/684 (5%)	
**Conservative treatment**				
0–4	274/464 (59%)	100/369 (27%)	95/366 (26%)	<0.001
5–10	67/464 (14%)	21/369 (6%)	16/366 (4%)	
>10	123/464 (27%)	248/369 (67%)	255/366 (70%)	

## Data Availability

Data are available upon request from the corresponding author.

## Supplementary Materials

The following supporting information can be downloaded at https://www.mdpi.com/article/10.3390/jcm15145441/s1: Table S1: Technical specification of propensity score matching procedures; Table S2: Covariate balance before and after propensity score matching: pre-pandemic versus during-pandemic; Table S3: Covariate balance before and after propensity score matching: pre-pandemic versus post-pandemic; Table S4: Hospitalization characteristics of women before and during pandemics (*n* = 1482); Table S5: Hospitalization characteristics of men before and during pandemics (*n* = 2082); Table S6: Hospitalization characteristics of women before and after pandemics (*n* = 1469;, Table S7: Hospitalization characteristics of men before and after pandemics (*n* = 1681); Table S8: Hospitalization characteristics of men before and after pandemics (*n* = 1681); Figure S1: Covariate balance before and after propensity score matching: pre-pandemic versus during-pandemic comparison; Figure S2: Covariate balance before and after propensity score matching: pre-pandemic versus post-pandemic comparison; Figure S3: Monthly admission trends from December 2013 to October 2023.

## References

[1] World Health Organization (2024). WHO Coronavirus (COVID-19) Dashboard.

[2] Willan J., King A.J., Djebbari F., Turner G.D.H., Royston D.J., Pavord S., Collins G.P., Peniket A. (2020). Assessing the impact of lockdown: Fresh challenges for the care of haematology patients in the COVID-19 pandemic. Br. J. Haematol..

[3] Harky A., Chiu C.M., Yau T.H.L., Lai S.H.D. (2020). Cancer patient care during COVID-19. Cancer Cell.

[4] Fersia O., Bryant S., Nicholson R., McMeeken K., Brown C., Donaldson B., Jardine A., Grierson V., Whalen V., Mackay A. (2020). The impact of the COVID-19 pandemic on cardiology services. Open Heart.

[5] Clarke J., Murray A., Markar S.R., Barahona M., Kinross J. (2020). New geographic model of care to manage the post-COVID-19 elective surgery aftershock in England: A retrospective observational study. BMJ Open.

[6] Birkmeyer J.D., Barnato A., Birkmeyer N., Bessler R., Skinner J. (2020). The impact of the COVID-19 pandemic on hospital admissions in the United States: Study examines trends in US hospital admissions during the COVID-19 pandemic. Health Aff..

[7] Kludacz-Alessandri M., Walczak R., Hawrysz L., Korneta P. (2021). The quality of medical care in the conditions of the COVID-19 pandemic, with particular emphasis on the access to primary healthcare and the effectiveness of treatment in Poland. J. Clin. Med..

[8] Mi B., Chen L., Xiong Y., Xue H., Zhou W., Liu G. (2020). Characteristics and early prognosis of COVID-19 infection in fracture patients. J. Bone Jt. Surg..

[9] Campbell E., Zahoor U., Payne A., Popova D., Welman T., Pahal G.S., Sadigh P. (2021). The COVID-19 pandemic: The effect on open lower limb fractures in a London major trauma centre—A plastic surgery perspective. Injury.

[10] Lim M.A., Mulyadi Ridia K.G., Pranata R. (2021). Epidemiological pattern of orthopaedic fracture during the COVID-19 pandemic: A systematic review and meta-analysis. J. Clin. Orthop. Trauma.

[11] Sephton B.M., Mahapatra P., Shenouda M., Ferran N., Deierl K., Sinnett T., Somashekar N., Sarraf K.M., Nathwani D., Bhattacharya R. (2021). The effect of COVID-19 on a Major Trauma Network. An analysis of mechanism of injury pattern, referral load and operative case-mix. Injury.

[12] Kumar A., Haider Y., Passey J., Khan R., Gaba S., Kumar M. (2021). Mortality predictors in COVID-19 positive patients with fractures: A systematic review. Bull. Emerg. Trauma.

[13] Murphy T., Akehurst H., Mutimer J. (2020). Impact of the 2020 COVID-19 pandemic on the workload of the orthopaedic service in a busy UK district general hospital. Injury.

[14] O’Hagan P., Drummond I., Lin D., Khor K.S., Vris A., Jeyaseelan L. (2021). Impact of the COVID-19 pandemic on the management of open fractures in a major trauma centre. J. Clin. Orthop. Trauma.

[15] Zhong H., Poeran J., Liu J., Wilson L.A., Memtsoudis S.G. (2021). Hip fracture characteristics and outcomes during COVID-19: A large retrospective national database review. Br. J. Anaesth..

[16] Egol K.A., Konda S.R., Bird M.L., Dedhia N., Landes E.K., Ranson R.A., Solasz S.J., Aggarwal V.K., Bosco J.A., Furgiuele D.L. (2020). Increased Mortality and Major Complications in Hip Fracture Care During the COVID-19 Pandemic: A New York City Perspective. J. Orthop. Trauma.

[17] Basile G. (2022). Remarks on the management of proximal femoral fractures in times of COVID-19 pandemic. Clin. Ter..

[18] Mamarelis G., Oduoza U., Chekuri R., Estfan R., Greer T. (2020). Mortality in Patients with Proximal Femoral Fracture During the COVID-19 Pandemic: A U.K. Hospital’s Experience. JBJS Open Access.

[19] von Elm E., Altman D.G., Egger M., Pocock S.J., Gøtzsche P.C., Vandenbroucke J.P. (2014). The Strengthening the Reporting of Observational Studies in Epidemiology (STROBE) Statement: Guidelines for reporting observational studies. Int. J. Surg..

[20] R Core Team (2023). R: A Language and Environment for Statistical Computing.

[21] Filip R., Gheorghita Puscaselu R., Anchidin-Norocel L., Dimian M., Savage W.K. (2022). Global challenges to public health care systems during the COVID-19 pandemic: A review of pandemic measures and problems. J. Pers. Med..

[22] Franco V.P., Gonçalves G.M., Fração O.C., Sungaila H.Y.F., Cocco L.F., Dobashi E.T. (2023). Evaluation of the epidemiology of exposed fractures before and during the covid-19 pandemic. Acta Ortop. Bras..

[23] Bronheim R.S., Humbyrd C.J. (2023). COVID-19 and the orthopaedic surgeon: Who gets redeployed?. J. Med. Ethics.

[24] Iyengar K., Vaish A., Vaishya R. (2020). Revisiting conservative orthopaedic management of fractures during COVID-19 pandemic. J. Clin. Orthop. Trauma.

[25] Raimondi S., Cammarata G., Testa G., Bellerba F., Galli F., Gnagnarella P., Iannuzzo M.L., Ricci D., Sartorio A., Sasso C. (2022). The impact of sport activity shut down during the COVID-19 pandemic on children, adolescents, and young adults: Was it worthwhile?. Int. J. Environ. Res. Public Health.

[26] Okereke I.C., Ramadan O., Sampalli S.R. (2021). The management of wrist fractures during COVID-19: A preliminary report. Cureus.

[27] Maliyappa C., Rupasinghe D., Iancu P., Mohamed M., Qazzaz L. (2023). Outcome analysis of adult distal radius fractures managed during COVID-19 pandemic. J. Orthop. Case Rep..

