# Supplementary material for: Lasting Shifts in Musculoskeletal Injuries Across Pre-, During-, and Post-Pandemic Periods: A Propensity Score-Matched Study
Source: J Clin Med. 2026 Jul 11;15(14):5441. doi: 10.3390/jcm15145441 (PMC13411168; doi:10.3390/jcm15145441)
Supplement: Supplementary file 1 [file jcm-15-05441-s001.zip › jcm-4344061-supplementary.pdf]

SUPPLEMENTARY FILE

Table S1. Technical specification of propensity score matching procedures.

Parameter	Pre-pandemic vs during-pandemic	Pre-pandemic vs post-pandemic
Comparison	Pre-pandemic vs during-pandemic	Pre-pandemic vs post-pandemic
Exposed group	During-pandemic admissions	Post-pandemic admissions
Control pool	Pre-pandemic admissions	Pre-pandemic admissions
Propensity score model	Logistic regression	Logistic regression
Variables included in PS model	Age, sex, hypertension, osteoporosis, diabetes mellitus	Age, sex, hypertension, osteoporosis, diabetes mellitus
Matching algorithm	Greedy nearest-neighbour propensity score matching	Greedy nearest-neighbour propensity score matching
Matching ratio	1:2	1:2
Replacement	No replacement	No replacement
Caliper	No caliper imposed	No caliper imposed
Estimand	ATT	ATT
Balance criterion	Absolute SMD < 0.10	Absolute SMD < 0.10
Exposed before matching	1188	1050
Control before matching	6175	6175
Exposed after matching	1188	1050
Control after matching	2376	2100
Unmatched exposed	0	0
Unmatched controls	3799	4075

ATT, average treatment effect in the treated; PS, propensity score; SMD, standardized mean difference. No caliper was imposed in the primary analysis.

Table S2. Covariate balance before and after propensity score matching: pre-pandemic versus during-pandemic.

	Before matching			After matching		
**Covariate**	**Before: exposed**	**Before: control**	**SMD before**	**After: exposed**	**After: control**	**SMD after**
Age	40.2 ± 20.3	43.9 ± 22.5	0.183	40.2 ± 20.3	40.6 ± 20.7	0.018
Sex: male	58.5%	58.2%	0.005	58.5%	58.4%	0.003
Hypertension	14.1%	17.1%	0.086	14.1%	14.9%	0.023
Osteoporosis	0.8%	1.1%	0.025	0.8%	0.4%	0.046
Diabetes mellitus	9.2%	6.0%	0.110	9.2%	9.1%	0.003

Continuous variables: mean ± SD; binary variables: percentages. SMD, standardized mean difference. Absolute SMD < 0.10 indicates acceptable balance. Maximum post-matching absolute SMD = 0.046.

Figure S1. Covariate balance before and after propensity score matching: pre-pandemic versus during-pandemic comparison.

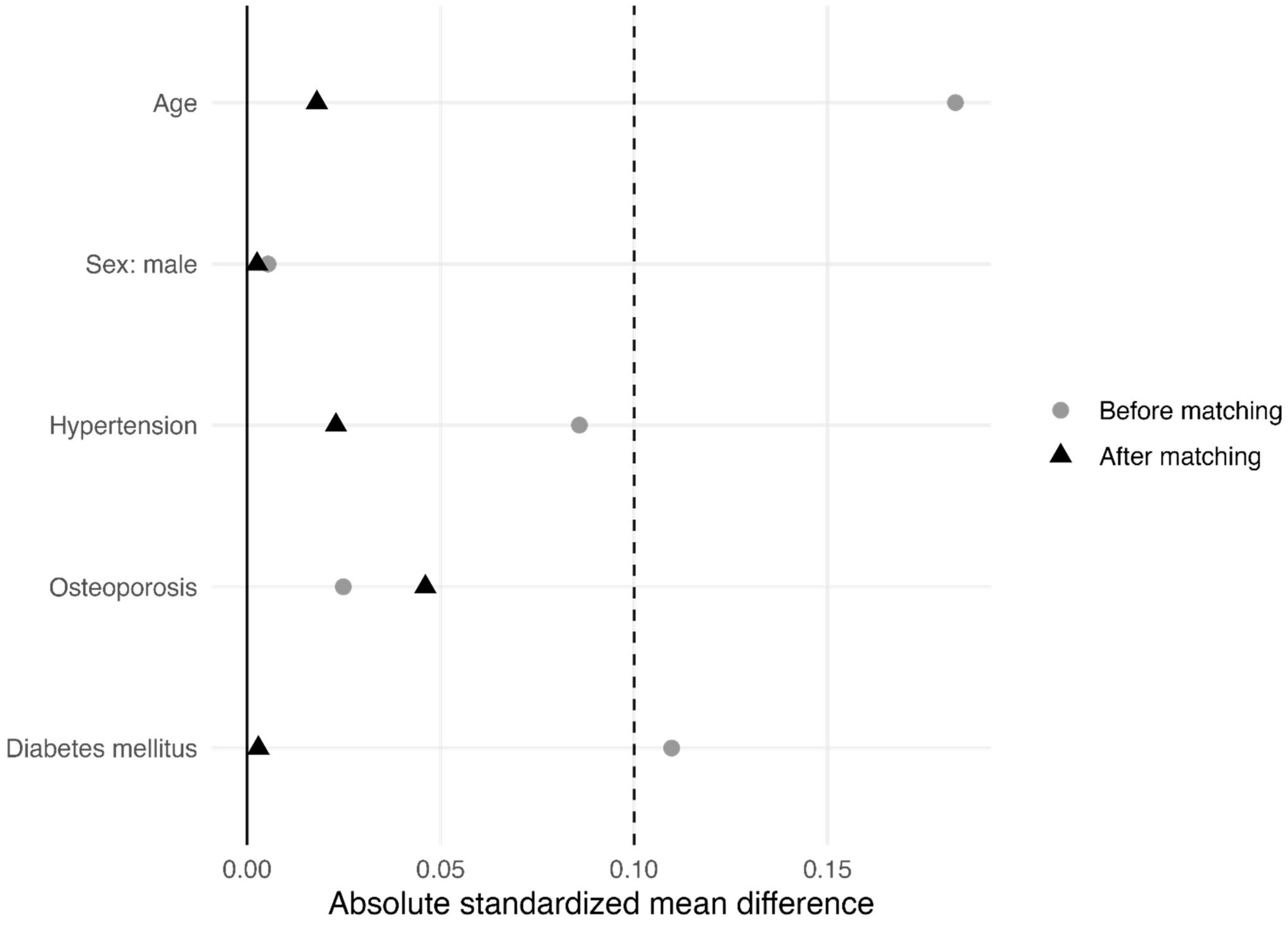

The Love plot shows absolute standardized mean differences for variables included in the propensity score model before and after matching. The vertical dashed line indicates the prespecified balance threshold of 0.10. Values below this threshold were considered indicative of acceptable covariate balance.

Table S3. Covariate balance before and after propensity score matching: pre-pandemic versus post-pandemic.

	Before matching			After matching		
**Covariate**	**Before: exposed**	**Before: control**	**SMD before**	**After: exposed**	**After: control**	**SMD after**
Age	42.8 ± 20.2	43.9 ± 22.5	0.058	42.8 ± 20.2	42.7 ± 20.1	0.002
Sex: male	53.2%	58.2%	0.100	53.2%	53.4%	0.004
Hypertension	17.3%	17.1%	0.005	17.3%	17.7%	0.009
Osteoporosis	1.9%	1.1%	0.061	1.9%	1.9%	0.000
Diabetes mellitus	6.0%	6.0%	0.000	6.0%	4.6%	0.060

Continuous variables: mean ± SD; binary variables: percentages. SMD, standardized mean difference. Absolute SMD < 0.10 indicates acceptable balance. Maximum post-matching absolute SMD = 0.060.

Figure S2. Covariate balance before and after propensity score matching: pre-pandemic versus post-pandemic comparison.

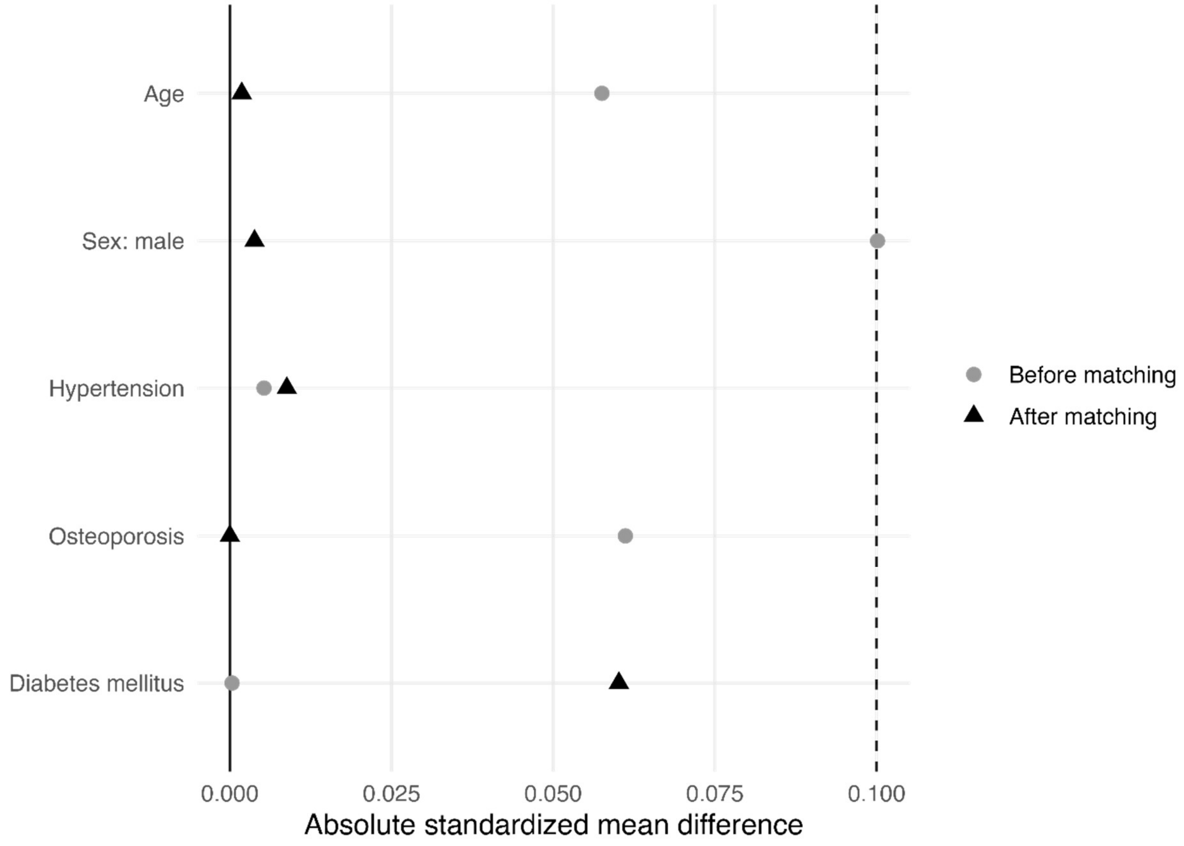

The Love plot shows

Absolute standardized mean differences for variables included in the propensity score model before and after matching. The vertical dashed line indicates the prespecified balance threshold of 0.10. Values below this threshold were considered indicative of acceptable covariate balance.

Table S4. Admission rates across pre-pandemic, during-pandemic and post-*pandemic periods.*

**Period**	**Start**	**End**	**Duration, months**	**Admissions, n**	**Admissions per month**	**Annualised admissions per year**	**IRR vs pre-pandemic (95% CI)**	**p value**
Pre-pandemic	2013-11-03	2020-03-03	76.0	6,175	81.3	975.1	Reference	
During-pandemic	2020-03-04	2022-05-13	26.3	1,188	45.1	541.7	0.56 (0.52 to 0.59)	<0.001
Post-pandemic	2022-05-14	2023-11-20	18.3	1,050	57.5	689.8	0.71 (0.66 to 0.76)	<0.001

IRR, incidence rate ratio. Admission rates were normalised by period duration because the study periods differed in length. IRRs were estimated using exact Poisson rate comparisons, with the pre-pandemic period as the reference.

Figure S3. Monthly admission trends from December 2013 to October 2023.

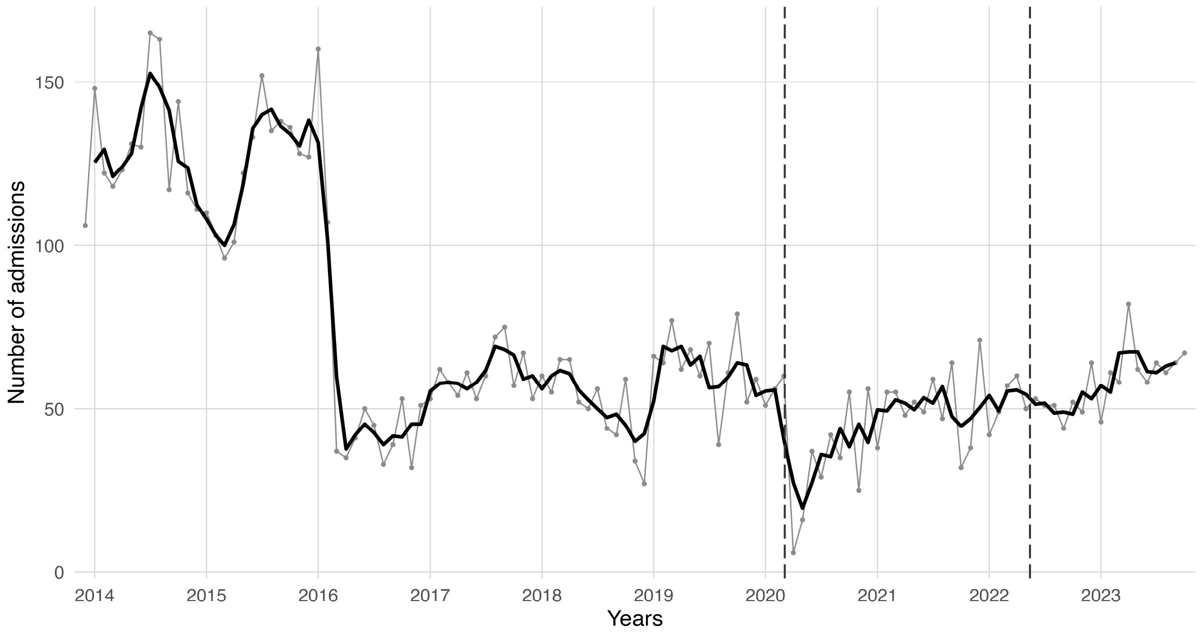

Monthly admission counts are shown across complete calendar months. Grey points and lines represent the observed monthly counts, while the black line represents a three-month moving average. Dashed vertical lines indicate the boundaries between the pre-pandemic, during-pandemic, and post-pandemic periods.

Table S5. Hospitalization characteristics of women before and during pandemics (N = 1,482).

	N	BEFORE N = 989[1]	DURING N = 493[1]	P[2]
**Type of injury**	1,475			**<0.001**
Primary		401 (41%)	26 (5%)	
Same limb re-injury		585 (59%)	463 (95%)	
**Workplace injury compensation plan**	1,482	53 (5%)	106 (22%)	**<0.001**
**Surgery**	1,482	770 (78%)	337 (68%)	**<0.001**
**Hospitalization length [days]**	1,482			**<0.001**
0-4		555 (56%)	305 (62%)	
5-10		278 (28%)	76 (15%)	
>10		156 (16%)	112 (23%)	
**Hospitalization length [days] – surgery**	1,107			**<0.001**
0-4		447 (58%)	262 (78%)	
5-10		248 (32%)	67 (20%)	
>10		75 (10%)	8 (2%)	
**Hospitalization length [days] – conservative treatment**	375			**<0.001**
0-4		108 (49%)	43 (28%)	
5-10		30 (14%)	9 (6%)	
>10		81 (37%)	104 (66%)	
**Localisation codes [ICD-10]**				
Dislocations, sprains, and strains of joints and ligaments of the pelvic region [S33]	1,482	7 (0.7%)	1 (0.2%)	0.28

	N	BEFORE N = 989[1]	DURING N = 493[1]	P[2]
Injuries of shoulder and upper arm [S40_S49]	1,482	120 (12%)	82 (17%)	**0.02**
Injuries of forearm [S50_S59]	1,482	231 (23%)	86 (17%)	**0.009**
Injuries of wrist and hand [S60_S69]	1,482	101 (10%)	38 (8%)	0.13
Injuries of hip and thigh [S70_S79]	1,482	166 (17%)	80 (16%)	0.82
Injuries of lower leg, including ankle and foot [S80_S89]	1,482	325 (33%)	189 (38%)	**0.04**
Injuries of ankle and foot region [S90_S99]	1,482	39 (4%)	17 (3%)	0.77
**Type of injury [ICD-10 codes]**				
Combined injuries [S40_50_60_70_80_90]	1,482	6 (0.6%)	2 (0.4%)	>0.99
Open wounds [S41_51_61_71_81_91]	1,482	12 (1%)	0 (0%)	**0.01**
Fractures [S42_52_62_72_82_92]	1,482	749 (76%)	366 (74%)	0.57
Dislocations and other injuries [S33_43_53_63_73_83_93]	1,482	167 (17%)	105 (21%)	**0.046**
Injuries to nerves [S44_54_64_74_84_94]	1,482	1 (0.1%)	1 (0.2%)	0.55
Injuries to muscles [S46_56_66_76_86_96]	1,482	42 (4%)	13 (3%)	0.14
Crush injuries [S48_58_68_78_88_98]	1,482	5 (0.5%)	1 (0.2%)	0.67
Other injuries [S49_59_69_79_89_99]	1,482	7 (1%)	5 (1%)	0.55
**Injury circumstances**	1,407			0.16
Life & Work		795 (86%)	426 (89%)	
Sport		131 (14%)	55 (11%)	
**Reason of sport related injury**				

	N	BEFORE<br>N = 989[1]	DURING<br>N = 493[1]	P[2]
Acrobatics, aerobics, dance, gymnastic	1,482	4 (0.4%)	1 (0.2%)	>0.99
Football and American football	1,482	10 (1%)	3 (1%)	0.56
Mountain climbing	1,482	2 (0.2%)	3 (0.6%)	0.34
Bicycle	1,482	38 (4%)	17 (3%)	0.77
Skiing	1,482	20 (2%)	18 (4%)	0.08
Other	1,482	38 (4%)	17 (3%)	0.77
Unspecified physical activity	1,482	7 (0.7%)	0 (0%)	0.1

[1]n / N (%)

[2]Fisher's test

Table S6. Hospitalization characteristics of men before and during pandemics (N = 2,082).

	N	BEFORE N = 1,387[1]	DURING N = 695[1]	P[2]
**Type of injury**	2,063			**<0.001**
Primary		681 (50%)	43 (6%)	
Same limb re-injury		693 (50%)	646 (94%)	
**Workplace injury compensation plan**	2,082	18 (1%)	147 (21%)	**<0.001**
**Surgery**	2,081	1,141 (82%)	482 (69%)	**<0.001**
**Hospitalization length [days]**	2,082			**<0.001**
0-4		843 (61%)	407 (59%)	
5-10		375 (27%)	111 (16%)	
>10		169 (12%)	177 (25%)	
**Hospitalization length [days] – surgery**	1,623			**<0.001**
0-4		677 (59%)	350 (73%)	
5-10		337 (30%)	99 (20%)	
>10		127 (11%)	33 (7%)	
**Hospitalization length [days] – conservative treatment**	458			**<0.001**
0-4		166 (68%)	166 (68%)	
5-10		37 (15%)	37 (15%)	
>10		42 (17%)	42 (17%)	
**Localisation codes [ICD-10]**				
Dislocations, sprains, and strains of joints and ligaments of the pelvic region [S33]	2,082	33 (2%)	13 (2%)	0.53

	N	BEFORE N = 1,387[1]	DURING N = 695[1]	P[2]
Injuries of shoulder and upper arm [S40_S49]	2,082	169 (12%)	70 (10%)	0.17
Injuries of forearm [S50_S59]	2,082	226 (16%)	124 (18%)	0.38
Injuries of wrist and hand [S60_S69]	2,082	240 (17%)	88 (13%)	**0.006**
Injuries of hip and thigh [S70_S79]	2,082	146 (11%)	90 (13%)	0.11
Injuries of lower leg, including ankle and foot [S80_S89]	2,082	470 (34%)	272 (39%)	**0.02**
Injuries of ankle and foot region [S90_S99]	2,082	103 (7%)	38 (5%)	0.1
**Type of injury [ICD-10 codes]**				
Combined injuries [S40_50_60_70_80_90]	2,082	9 (0.6%)	2 (0.3%)	0.35
Open wounds [S41_51_61_71_81_91]	2,082	79 (5.7%)	6 (0.9%)	**<0.001**
Fractures [S42_52_62_72_82_92]	2,082	905 (65%)	451 (65%)	0.88
Dislocations and other injuries [S33_43_53_63_73_83_93]	2,082	250 (18%)	143 (21%)	0.17
Injuries to nerves [S44_54_64_74_84_94]	2,082	3 (0.2%)	2 (0.3%)	>0.99
Injuries to muscles [S46_56_66_76_86_96]	2,082	109 (7.9%)	53 (7.6%)	0.93
Amputations [S47_57_67_77_87_97]	2,082	2 (0.1%)	2 (0.3%)	0.60
Crush injuries [S48_58_68_78_88_98]	2,082	26 (1.9%)	24 (3.5%)	**0.03**
Other injuries [S49_59_69_79_89_99]	2,082	4 (0.3%)	12 (1.7%)	**<0.001**
**Injury circumstances**	1,997			
Life & Work		1,064 (81%)	584 (86%)	**0.006**
Sport		252 (19%)	97 (14%)	

	N	BEFORE<br>N = 1,387[1]	DURING<br>N = 695[1]	P[2]
**Reason of sport related injury**				
Football and American football	2,082	80 (6%)	24 (3%)	**0.03**
Mountain climbing	2,082	1 (<0.1%)	1 (0.1%)	>0.99
Surfing, windsurfing, skateboarding, wakeboarding	2,082	3 (0.2%)	0 (0%)	0.56
Bicycle	2,082	67 (5%)	27 (4%)	0.37
Skiing	2,082	29 (2%)	16 (2%)	0.75
Other	2,082	67 (5%)	27 (4%)	0.37
Unspecified physical activity	2,082	10 (0.7%)	0 (0%)	**0.04**

[1]n / N (%)

[2]Fisher's test

Table S7. Hospitalization characteristics of women before and after pandemics (N = 1.469).

	N	BEFORE, N = 978[1]	AFTER, N = 491[1]	P[2]
**Type of injury**	1,461			**<0.001**
Primary		414 (42%)	22 (5%)	
Same limb re-injury		561 (58%)	464 (95%)	
**Workplace injury compensation plan**	1,469	49 (5%)	143 (29%)	**<0.001**
**Surgery**	1,469	777 (79%)	299 (61%)	**<0.001**
**Hospitalization length [days]**	1,469			**<0.001**
0-4		519 (53%)	280 (57%)	
5-10		307 (31%)	58 (12%)	
>10		152 (16%)	153 (31%)	
**Hospitalization length [days] – surgery**	1,076			**<0.001**
0-4		425 (55%)	236 (79%)	
5-10		270 (35%)	49 (16%)	
>10		82 (10%)	14 (5%)	
**Hospitalization length [days] – conservative treatment**	393			**<0.001**
0-4		94 (47%)	44 (23%)	
5-10		37 (18%)	9 (5%)	
>10		70 (35%)	139 (72%)	
**Localisation codes [ICD-10]**				
Dislocations, sprains, and strains of joints and ligaments of the pelvic region [S33]	1,469	8 (0.8%)	3 (0.6%)	0.76

	N	BEFORE, N = 978[1]	AFTER, N = 491[1]	P[2]
Injuries of shoulder and upper arm [S40_S49]	1,469	115 (12%)	48 (10%)	0.29
Injuries of forearm [S50_S59]	1,469	211 (22%)	69 (14%)	**<0.001**
Injuries of wrist and hand [S60_S69]	1,469	97 (10%)	39 (8%)	0.25
Injuries of hip and thigh [S70_S79]	1,469	176 (18%)	95 (19%)	0.52
Injuries of lower leg, including ankle and foot [S80_S89]	1,469	329 (34%)	224 (46%)	**<0.001**
Injuries of ankle and foot region [S90_S99]	1,469	42 (4%)	13 (3%)	0.14
**Type of injury [ICD-10 codes]**				
Combined injuries [S40_50_60_70_80_90]	1,469	7 (0.7%)	1 (0.2%)	0.28
Open wounds [S41_51_61_71_81_91]	1,469	16 (1.6%)	2 (0.4%)	**0.046**
Fractures [S42_52_62_72_82_92]	1,469	731 (75%)	350 (71%)	0.17
Dislocations and other injuries [S33_43_53_63_73_83_93]	1,469	168 (17%)	120 (24%)	**0.001**
Injuries to nerves [S44_54_64_74_84_94]	1,469	1 (0.1%)	2 (0.4%)	0.26
Injuries to muscles [S46_56_66_76_86_96]	1,469	45 (6%)	9 (2%)	**0.008**
Amputations [S47_57_67_77_87_97]	1,469	0 (0%)	4 (0.8%)	**0.01**
Crush injuries [S48_58_68_78_88_98]	1,469	5 (0.5%)	2 (0.4%)	>0.99
Other injuries [S49_59_69_79_89_99]	1,469	5 (0.5%)	1 (0.2%)	0.67
**Injury circumstances**	1,388			**0.03**
Life & Work		777 (85%)	428 (90%)	

	N	BEFORE, N = 978[1]	AFTER, N = 491[1]	P[2]
Sport		133 (15%)	50 (10%)	
**Reason of sport related injury**				
Acrobatics, aerobics, dance, gymnastic	1,469	3 (0.3%)	0 (0%)	0.56
Football and American football	1,469	13 (1.3%)	1 (0.2%)	**0.04**
Mountain climbing	1,469	4 (0.4%)	4 (0.8%)	0.45
Surfing, windsurfing, skateboarding, wakeboarding	1,469	1 (0.1%)	1 (0.2%)	>0.99
Bicycle	1,469	37 (4%)	10 (2%)	0.08
Skiing	1,469	24 (2%)	18 (4%)	0.19
Other	1,469	37 (4%)	10 (2.%)	0.08
Unspecified physical activity	1,469	4 (0.4%)	0 (0%)	0.31

[1]n / N (%)

[2]Fisher's test

Table S8. Hospitalization characteristics of men before and after pandemics (N = 1,681).

	N	BEFORE, N = 1,122[1]	AFTER, N = 559[1]	P[2]
**Type of injury**	1,667			**<0.001**
Primary		540 (49%)	28 5%)	
Same limb re-injury		573 (51%)	526 (95%)	
**Workplace injury compensation plan**	1,681	10 (1%)	114 (20%)	**<0.001**
**Surgery**	1,680	937 (84%)	385 (69%)	**<0.001**
**Hospitalization length [days]**	1,681			**<0.001**
0-4		675 (60%)	338 (60%)	
5-10		312 (28%)	85 (15%)	
>10		135 (12%)	136 (25%)	
**Hospitalization length [days] – surgery**	1,322			**<0.001**
0-4		548 (58%)	287 (75%)	
5-10		283 (31%)	78 (20%)	
>10		106 (11%)	20 (5%)	
**Hospitalization length [days] – conservative treatment**	358			**<0.001**
0-4		127 (69%)	51 (29%)	
5-10		28 (15%)	7 (4%)	
>10		29 (16%)	116 (67%)	
**Localisation codes [ICD-10]**				
Dislocations, sprains, and strains of joints and ligaments of the pelvic region [S33]	1,681	31 (3%)	7 (1%)	0.06

	N	BEFORE, N = 1,122[1]	AFTER, N = 559[1]	P[2]
Injuries of shoulder and upper arm [S40_S49]	1,681	135 (12%)	62 (11%)	0.63
Injuries of forearm [S50_S59]	1,681	167 (15%)	95 (17%)	0.28
Injuries of wrist and hand [S60_S69]	1,681	209 (19%)	55 (10%)	**<0.001**
Injuries of hip and thigh [S70_S79]	1,681	125 (11%)	63 (11%)	0.93
Injuries of lower leg, including ankle and foot [S80_S89]	1,681	379 (34%)	243 (43%)	**<0.001**
Injuries of ankle and foot region [S90_S99]	1,681	76 (7%)	34 (6%)	0.68
**Type of injury [ICD-10 codes]**				
Combined injuries [S40_50_60_70_80_90]	1,681	9 (0.8%)	1 (0.2%)	0.18
Open wounds [S41_51_61_71_81_91]	1,681	70 (6%)	4 (1%)	**<0.001**
Fractures [S42_52_62_72_82_92]	1,681	704 (63%)	336 (60%)	0.31
Dislocations and other injuries [S33_43_53_63_73_83_93]	1,681	220 (20%)	150 (27%)	**<0.001**
Injuries to nerves [S44_54_64_74_84_94]	1,681	2 (0.2%)	6 (1.1%)	**0.02**
Injuries to muscles [S46_56_66_76_86_96]	1,681	95 (9%)	42 (8%)	0.57
Amputations [S47_57_67_77_87_97]	1,681	1 (<0.1%)	0 (0%)	>0.99
Crush injuries [S48_58_68_78_88_98]	1,681	18 (2%)	17 (3%)	0.07
Other injuries [S49_59_69_79_89_99]	1,681	3 (0.3%)	3 (0.5%)	0.41
**Injury circumstances**	1,595			**0.03**
Life & Work		844 (80%)	455 (84%)	

	**N**	**BEFORE**, N = 1,122[1]	**AFTER**, N = 559[1]	**P**[2]	
Sport		212 (20%)	84 (16%)		
**Reason of sport related injury**					
Football and American football		1,681	73 (7%)	27 (5%)	0.19
Mountain climbing		1,681	1 (<0.1%)	2 (0.4%)	0.26
Surfing, windsurfing, skateboarding, wakeboarding		1,681	3 (0.3%)	0 (0%)	0.56
Bicycle		1,681	56 (5%)	27 (5%)	>0.99
Skiing		1,681	24 (2%)	12 (2%)	>0.99
Other		1,681	56 (5%)	27 (5%)	>0.99
Unspecified physical activity		1,681	10 (0.9%)	0 (0%)	**0.04**

[1]n / N (%)

[2]Fisher's test
